# Complete Genome Sequences of Plasmid-Bearing *Campylobacter coli* and *Campylobacter jejuni* Strains Isolated from Retail Chicken Liver

**DOI:** 10.1128/genomeA.01350-17

**Published:** 2017-12-07

**Authors:** Daya Marasini, Mohamed K. Fakhr

**Affiliations:** Department of Biological Science, The University of Tulsa, Tulsa, Oklahoma, USA

## Abstract

Complete genome sequences of *Campylobacter coli* strains WA333, YF2105, BG2108, MG1116, and BP3183 and *Campylobacter jejuni* strain IF1100 isolated from retail chicken liver showed the presence of 1,841,551-, 1,687,232-, 1,695,638-, 1,665,146-, 1,695,360-, and 1,744,171-bp circular chromosomes, respectively. These isolates also contained plasmids ranging in size from 5,209 to 55,122 bp.

## GENOME ANNOUNCEMENT

A high prevalence (77%) of *Campylobacter jejuni* and *Campylobacter coli* has been reported in retail chicken and beef liver samples (1, 2). A multistate outbreak of *C. jejuni* associated with undercooked chicken livers occurred in the United States in 2012 (3). A chicken liver-associated foodborne outbreak caused by *Campylobacter* spp. was reported in Liverpool, United Kingdom (4). Another study in the United Kingdom showed high survival rates of *Campylobacter* spp. in undercooked chicken livers at restaurants (5). Multilocus sequence typing showed similarities between *Campylobacter* strains isolated from chicken livers and those causing human illnesses (6, 7). The prevalence of plasmids has been reported to be high in *Campylobacter* strains isolated from retail chicken liver (8). Other foodborne pathogens, such as *Staphylococcus aureus*, were also reported to be prevalent in retail beef and chicken livers (9, 10).

Here, we announce the complete genome sequences of five *C. coli* and one *C. jejuni* chicken liver isolates. These isolates were previously isolated from retail chicken liver (1). The genomic and plasmid DNA was isolated from a 72-h liquid culture using a DNeasy blood and tissue kit (Qiagen, Inc., Valencia, CA) and plasmid midikit (Qiagen, Inc.). The genome sequencing was performed using the Illumina MiSeq desktop sequencer by applying the Illumina v2 reagent kit. Library preparation was performed using the Nextera XT sample preparation kit (Illumina, Inc., San Diego, CA). Assembly of the chromosomes and plasmids was carried out using CLC genomic workbench and its microbial genome finishing module (Qiagen, Inc.).

The genomes of *C. coli* WA333, YF2105, BG2108, MG1116, and BP3183 and *C. jejuni* IF1100 contained one full circular chromosome each with 1,841,551, 1,687,232, 1,695,638, 1,665,146, 1,695,360, and 1,744,171 bp and 1,965, 1,864, 1,878, 1,836, 1,836, and 1,878 genes, respectively. All of the isolates contained plasmids ranging in size from 5 kb to 55 kb. Plasmids of larger sizes were previously reported in *Campylobacter* spp. isolated from various retail meats (8, 11–13). The *C. coli* WA333 strain contained one 25,058-bp plasmid (pCCDM33S) with 30 coding sequences (CDS) coding for conjugal transfer and some virulence proteins. *C. coli* YF2105 contained two plasmids, pCCDM105L (46,193 bp) and pCCDM105S (25,284 bp), with 53 and 30 CDS, respectively. *C. coli* BG2108 contained two plasmids, pCCDM108L (46,186 bp) and pCCDM108S (25,286 bp), having 53 and 30 CDS, respectively. *C. coli* MG1116 contained two plasmids, pCCDM116L (45,633 bp) and pCCDM116S (24,874 bp), with 53 and 29 CDS, respectively. *C. coli* BP3183 contained one plasmid, pCCDM183 (55,122 bp), with multidrug-resistance genes similar to those in the megaplasmid present in *C. jejuni* T1-21 (11). *C. jejuni* IF1100 contained one small plasmid, pCJDM100 (5,209 bp), with 6 CDS containing a replication initiator protein. This strain contained a type VI secretion system in the chromosome. Most of the plasmids larger than 44 kb contained a type IV secretion system and tetracycline resistance genes. The G+C contents of these six isolates ranged from 28 to 33%.

### Accession number(s).

The genome sequences of the *C. coli* and *C. jejuni* strains reported here have been deposited in GenBank under the accession numbers listed in Table 1.

**TABLE 1:** Accession numbers and sizes of *Campylobacter* genomes sequenced in this study

Strain name	GenBank accession no.^a^	Sequence length (bp)
WA333	CP017873	1,841,551
	CP017874	25,058
YF2105	CP017865	1,687,232
	CP017866	46,193
	CP017867	25,284
BG2108	CP017878	1,695,638
	CP017879	46,186
	CP017880	25,286
MG1116	CP017868	1,665,146
	CP017869	45,633
	CP017870	24,874
BP3183	CP017871	1,695,360
	CP017872	55,122
IF1100	CP017863	1,744,171
	CP017864	5,209

a For each isolate, chromosomes are listed first, followed by the plasmid(s).
